# Effects of motivation on reward and attentional networks: an fMRI study

**DOI:** 10.1002/brb3.80

**Published:** 2012-09-23

**Authors:** Iliyan Ivanov, Xun Liu, Suzanne Clerkin, Kurt Schulz, Karl Friston, Jeffrey H Newcorn, Jin Fan

**Affiliations:** 1 Department of Psychiatry, Mount Sinai School of MedicineOne Gustave L. Levy Place, New York, New York, 10029; 2 Key Laboratory of Behavior Science, Institute of Psychology, Chinese Academy of ScienceBeijing, China; 3 Wellcome Center for Neuroimaging, University College London12 Queen Square, London, WC1N 3BG, United Kingdom; 4 Department of Psychology, Queens College, City University of New YorkFlushing, New York, 11367

**Keywords:** Attention, brain reward system, fMRI, motivation, neuroimaging, neuroscience

## Abstract

Existing evidence suggests that reward and attentional networks function in concert and that activation in one system influences the other in a reciprocal fashion; however, the nature of these influences remains poorly understood. We therefore developed a three-component task to assess the interaction effects of reward anticipation and conflict resolution on the behavioral performance and the activation of brain reward and attentional systems. Sixteen healthy adult volunteers aged 21–45 years were scanned with functional magnetic resonance imaging (fMRI) while performing the task. A two-way repeated measures analysis of variance (ANOVA) with cue (reward vs. non-reward) and target (congruent vs. incongruent) as within-subjects factors was used to test for main and interaction effects. Neural responses to anticipation, conflict, and reward outcomes were tested. Behaviorally there were main effects of both reward cue and target congruency on reaction time. Neuroimaging results showed that reward anticipation and expected reward outcomes activated components of the attentional networks, including the inferior parietal and occipital cortices, whereas surprising non-rewards activated the frontoinsular cortex bilaterally and deactivated the ventral striatum. In turn, conflict activated a broad network associated with cognitive control and motor functions. Interaction effects showed decreased activity in the thalamus, anterior cingulated gyrus, and middle frontal gyrus bilaterally when difficult conflict trials (e.g., incongruent targets) were preceded by reward cues; in contrast, the ventral striatum and orbitofrontal cortex showed greater activation during congruent targets preceded by reward cues. These results suggest that reward anticipation is associated with lower activation in attentional networks, possibly due to increased processing efficiency, whereas more difficult, conflict trials are associated with lower activity in regions of the reward system, possibly because such trials are experienced as less rewarding.

## Introduction

Motivational states are widely thought to modulate the salience of behavioral goals and to influence attention and behavioral control in relation to goal pursuit and completion (Kruglanski et al. 2002). While our understanding of the interaction between motivation and cognitive control has grown (Small et al. 2005; Locke and Braver 2008; Mohanty et al. 2008; Engelmann et al. 2009; Pessoa 2009; Beck et al. 2010; Daniel and Pollmann 2010; Padmala and Pessoa 2010), the neurobiological mechanisms by which motivation affects the ability to control attention to task demands and influence task performance remain poorly characterized. Animal studies suggest that structures involved in attention, such as the lateral intraparietal area, also process information related to reward contingencies (Platt and Glimcher 1999; Sugrue et al. 2004) and may be involved in the integration of attentional control and motivation (Bendiksby and Platt 2006). Accordingly, recent neuroimaging studies have begun to probe the neural correlates of the interaction between motivation and cognitive control in humans (Small et al. 2005; Mohanty et al. 2008; Savine and Braver 2010; Padmala and Pessoa 2011).

One conceptual framework speculates that motivation may enhance performance by “energizing” and “speeding-up” processing. Others have suggested that interactions between motivation and performance are more nuanced and that reward incentives may have selective effects on cognitive processes. The latter thesis is supported by reports showing that motivation to obtain rewards may reduce conflict-related activation in the medial prefrontal cortex and the anterior cingulate cortex (ACC) (Padmala and Pessoa 2011) and that it may enhance cue-related activation in the dorsolateral prefrontal cortex (DLPFC), which, in turn, optimizes performance (Savine and Braver 2010). Furthermore, these types of interaction seem to be associated with amplification (Egner and Hirsch 2005) and/or improved filtering of task-irrelevant information (Polk et al. 2008). Conversely, potentially deleterious effects of motivation for rewards on performance have been suggested by reports of prolonged stop-signal reaction time and significant inhibition of blood oxygenation level-dependent (BOLD) activation in the right inferior frontal gyrus, the left precentral gyrus, and bilateral putamen in relation to rewards (Padmala and Pessoa 2010). A more detailed examination of the interactions between the effects of motivation and cognitive control on performance is important for two main reasons: (i) to elucidate the neurobiological mechanisms associated with the interaction between motivation and cognitive control; and (ii) to advance the understanding of the interaction between motivation and diminished behavioral control as a central feature of clinical syndromes, such as attention deficit/hyperactivity disorder, obsessive–compulsive disorder, and drug abuse disorders (Garavan and Stout 2005; Li et al. 2008; Chambers et al. 2009).

Two types of motivational processes have been proposed. One process is oriented toward potentially rewarding outcomes, and the other is oriented toward potentially aversive outcomes (Lang et al. 1998; Elliot and Covington 2001). These processes are thought to be linked to neurobiological systems that are sensitive to rewards and punishments, respectively (Elliot and Thrash 2002). Brain regions involved in the processing of rewards and punishment include ventral striatum, orbitofrontal cortex (OFC), ACC, and DLPFC among others (Spielberg et al. 2012). These systems influence attention to rewarding and punishing stimuli, as well as behavioral responses to motivationally relevant stimuli (Elliot and Thrash 2002). Individual differences in the activity and/or reactivity of these systems are heritable, present early in life, and stable over the lifespan (Clark et al. 1994; Elliot and Thrash 2002).

Interactions between motivation and cognitive control can be assessed by a variety of methods. One is to measure task performance under different conditions (i.e., with vs. without reward incentives) and to compare differences in performance. This method is illustrated by studies using tasks that engage executive functions, such as attention (Engelmann et al. 2009), information-integration learning (Daniel and Pollmann 2010), working memory (Beck et al. 2010), or response inhibition (Small et al. 2005; Locke and Braver 2008). We have adopted an alternative approach by combining a validated reward paradigm, the Monetary Incentive Delay (MID) task (Knutson et al. 2000), with the Erickson flanker task (Eriksen and Eriksen 1974). The MID consists of graded reward cues, a target to which the subjects must respond as fast as possible by pressing a button, and reward outcomes that include monetary gain, no gain, or loss. The participants are instructed that the different reward outcomes depend on the quickness of their response; however, in reality, the task outcomes are predetermined so that each subject experiences an equal percentage of win, no win, and loss trials. For the purpose of this study, we substituted the simple reaction time (RT) response from the MID with a flanker task, in which participants have to respond to a center arrow flanked by two arrows pointing in either the same or the opposite direction. The MID has been reported to consistently elicit activation in the brain regions associated with both attention and reward, for example, frontoinsular cortex, caudate, putamen, the medial prefrontal cortex (Knutson et al. 2000; Knutson et al. 2004; Bjork and Hommer 2007; Knutson and Wimmer 2007), nucleus accumbens (NAcc) (Knutson et al. 2000; Cooper et al. 2009), as well as the ACC (Linke et al. 2010). The flanker task has consistently activated brain regions associated with cognitive control, such as the ACC, DLPFC (Fan et al. 2003; Brown 2009; Morishima et al. 2010), and left superior and middle frontal gyri (Zhu et al. 2010).

The effects of motivation on reducing conflict have been addressed by others (Padmala and Pessoa 2011). Our task, called the Anticipation, Conflict, and Reward (ACR) task, supports lower reward probabilities combined with high attentional demand in relation to alternative tasks. Moreover, the ACR task includes a surprising non-reward component that allows one to assess violation of reward expectations. The ACR task is shorter than similar tasks and is particularly suited for use in youths. We have found this task particularly useful when studying young children at risk for later addiction (Ivanov et al., 2012). Crucially, the ACR design allows one to assess the effects of cognitive demands on reward processing, through interaction effects, framed in terms of task components. The current study used the ACR task and functional magnetic resonance imaging (fMRI) to assess the interactions between anticipation, cognitive demand, and reward processing, under expected reward and unexpected reward outcomes. On the basis of the available literature (Engelmann et al. 2009; Pessoa and Engelmann 2010), we predicted that motivation (reward anticipation) would modulate processing in attentional regions such as frontoparietal cortex, and specifically decrease activity in regions associated with conflict resolution, such as the ACC. Furthermore, considering findings suggesting that activation in the ventral striatum may be inversely influenced by the degree of cognitive demand for a given task (Botvinick et al. 2009), we hypothesized that conflict would be associated with reduced activation in the reward network, including the ventral striatum and the OFC.

## Methods

### Participants

Sixteen healthy right-handed adults (six females) aged 21–45 years (mean = 30.63, SD = 7.44) participated in the study over two visits. During the first visit, all participants signed an informed consent form approved by the Mount Sinai School of Medicine Institutional Review Board. Participants also received a physical exam, an electrocardiogram, and blood pressure readings and were screened for current or past history of head injuries, neurological or cardiovascular disease, other systemic illness, and contraindications for MRI. In addition, board certified psychiatrists (J. H. N. and I. I.) performed a mental status exam to screen for a current or past psychiatric history using the screen section of the Structured Clinical Interview for *DSM-IV* (Spitzer et al. 1992). Participants completed the Symptom Checklist-90-R (SCL-90-R)(Cyr et al. 1988), the Michigan Assessment-Screening Test/Alcohol-Drug (MAST-AD)(Westermeyer et al. 2004), and Conners' Adult ADHD Rating Scale – Self-Report: Long Version CAARS (Conners 2000). The Matrix Reasoning and Vocabulary subtests of the Wechsler Abbreviated Scale of Intelligence (WASI) (Ryan et al. 2003) were administered to estimate Full Scale IQ. A T-score of 1.5 SD on the CAARS and total ADHD Symptoms index (indicating the possibility of ADHD) and the SCL-90 Total Severity Index (indicating the possibility of other psychopathology) above age and gender means (i.e., >65), or an estimated IQ <80 (indicating low cognitive capacity) were used as exclusion criteria. Suspected current drug abuse, indicated by a MAST-AD score >5, was also exclusionary. Sample characteristics are presented in Table 1.

**Table 1:** Sample characteristics

	Mean	SD
Age	30.63	±7.44
MAST^1^	1.81	±4.49
CAARS–ADHD Index^2^	40.81	±5.66
SCL-90 Somatization^2^	40.00	±40.92
SCL-90 Obsessive–compulsive^2^	40.91	±9.13
SCL-90 Interpersonal sensitivity^2^	41.67	±9.75
SCL-90 Depression^2^	42.33	±10.51
SCL-90 Anxiety^2^	36.58	±4.25
SCL-90 Hostility^2^	44.00	±7.36
SCL-90 Phobic anxiety^2^	44.08	±5.71
SCL-90 Paranoid ideation^2^	42.42	±3.82
SCL-90 Psychoticism^2^	43.00	±7.45
SCL-90 Global severity index^2^	37.91	±9.04
SCL-90 Positive symptom total^2^	41.42	±12.57
SCL-90 Positive symptom distress index^2^	48.83	±8.75

1 Results presented in raw scores; MAST raw scores >5 are considered abnormal.

2 Results presented in T-scores; in this study scores on CAARS and SCL-90 that were >1.5 SD above the mean were considered abnormal.

It has been generally accepted that an fMRI study with 16 participants is adequate to provide sufficient power to detect statistically significant changes in brain activation (Desmond and Glover 2002; Murphy and Garavan 2004). Furthermore, a recent report that specifically focused on the calculation of power analyses in fMRI protocols suggests that the number of subjects needed to achieve 80% is related to the length of the scan time. For instance, tasks that require scan time of 5–6 min will need a sample of 22–24 subjects, whereas tasks with scan time of 13 min will achieve similar power with a sample of 17 subjects (Mumford and Nichols 2008). As the ACR is 24 min in length, it is very likely that 16 subjects are sufficient to detect meaningful differences in regional activation.

### Procedures

The fMRI scans were performed during a second study visit, approximately 14 days following the first visit. Participants practiced one block of the task on a desktop computer prior to the scan. The length of the scanning procedure was 35–40 min.

### ACR paradigm

**Figure 1:**
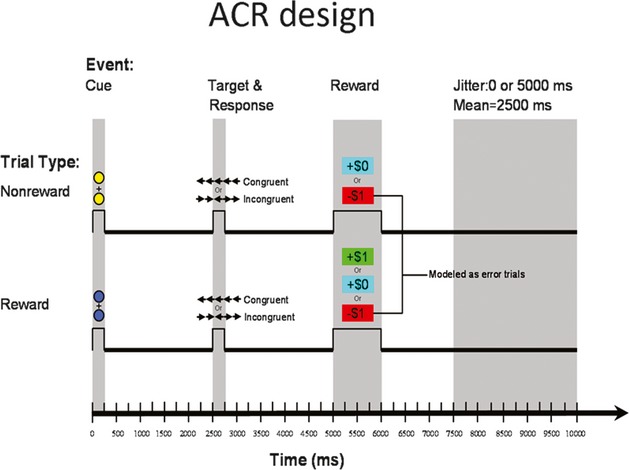
Anticipation, conflict, and reward task. This schematic shows the temporal relationship between the cue, target, and outcome components of the ACR task. Sixty-four reward cues (blue circle) and 64 non-reward cues (yellow circle), as well as 64 congruent targets and 64 incongruent targets are randomly presented during the four sessions of the task. The outcome is performance dependent: subjects must respond as quickly as possible by pushing a button with their left or right index finger that corresponds to the direction of the to the center arrow of the flanker. If the response is correct, there is 50% chance of reward in the amount of $1 (green square); slow and/or incorrect responses result in $1 loss (red square). In non-rewarding trials the reward is omitted.

The ACR is a hybrid task based on the MID (Knutson et al. 2001), in which a conflict manipulation is added to the reward anticipation and outcome components of the original task (Fig. 1). Specifically, the simple RT task in the MID is replaced with a flanker task from the Attention Network Test (Knutson and Wimmer 2007). Thus, the ACR provides three distinct probes of reward anticipation, conflict resolution, and reward outcomes. In the context of fMRI, the ACR task is designed with a fixed rather than a jittered cue-target interval to minimize the length of each compound trial. This enables hemodynamic responses to be modeled purely in terms of task and stimulus-related components and avoids assumptions about delay period activity or sustained neuronal responses. Previous studies have used a jittered cue-target interval to ensure a reasonably efficient deconvolution of the hemodynamic response to cues and targets; however, this deconvolution rests upon assumptions about sustained neuronal responses and reduces the overall efficiency for detecting event-related responses. In contrast, the ACR task relies upon task analysis and design to orthogonalize the task components. We have found that a fixed 2250-msec cue-target interval provides efficient estimates of cue and target-related response components (Clerkin et al. 2009; Schulz et al. 2011).

The ACR protocol comprises four (6 min and 20 sec) 32-trial blocks including 30-sec fixations at the beginning and the end of each block. All trials begin with a cue presented at fixation for 250 msec, followed by a 2250-msec fixation period. A target is then displayed at fixation for 250 msec, followed by 2250-msec fixation period. Reward outcome is then displayed at fixation for 1000 ms, followed by 1500-msec fixation period. The intertrial interval is jittered from 0 to 5000 msec, with a mean of 2500 msec in each block. The average length of each trial is therefore 10 sec (Fig. 1).

The task contains two trial types: non-reward and reward trials. Non-reward trials begin with a yellow circle indicating that non-reward will be delivered, followed by a target, which is a central arrowhead, surrounded by double arrowheads on each side that are either congruent or incongruent in direction. Subjects must respond in the direction of the central arrowhead as soon as possible, while ignoring the flanker arrowheads. The congruent versus incongruent flankers are counterbalanced within each block. The outcome for a correct response in a non-reward trial is $0, which is displayed in a light blue square. Reward trials begin with a blue circle indicating rewards are available, followed by a target (as described previously). The outcome for a correct response in a reward trial is +$1, which is displayed in a green square. There is a 50% probability of receiving a reward (i.e., only half of the 64 reward cue trials are rewarded); therefore, the maximum win for each block is $8, and the maximum win for the whole task is $32. The outcome for an incorrect or delayed response is −$1 (displayed in a red square); mistakes on non-rewarding trials are also punished. The punishment or lost revenue is subtracted from the sum already gained or added as negative balance. The running total of winnings/losses is presented at the end of each block of the task. The monetary reward value associated with the ACR is virtual and not real – the reimbursement given to participants was the same (e.g., $100 per session) – and subjects were aware of this before scanning. This design corresponds to a nested factorial design with three factors: anticipation (reward vs. non-reward cue), conflict (congruent vs. incongruent flankers), and reward outcomes.

Reward outcomes are defined in relation to reward cues as (i) expected reward–reward cues followed by $1 win for correct responses, (ii) expected non-reward–non-reward cues followed by $0 for correct responses, and (iii) surprising non-reward–reward cue followed by $0 for correct responses. The 32 trials in each block were evenly divided into non-reward and reward trials and are counterbalanced within each block. Participants were told that if they respond correctly to the target that followed a reward cue, they can receive a one dollar reward (detailed instructions are presented in Supporting Information). They were also instructed that if they failed to respond or if the response was incorrect or slow, a dollar would be taken away. Slow responses were defined as button presses slower than 750 msec.

### Image acquisition

All participants were scanned on a 3.0 Tesla Siemens Allegra (Siemens Medical Systems, Erlangen, Germany) head-dedicated MRI scanner using a high-performance head gradient system. Participants were fitted with headphones and their heads were stabilized with firm foam padding. Stimuli were projected via an Super Video Graphics Array system onto a rear-projection screen mounted at the head of the magnet bore. Subjects viewed the stimuli through a mirror on the head coil positioned above their eyes.

Scan sessions began with shimming and sagittal localization. Next, a high-resolution T2-weighted anatomical volume of the brain was acquired with a turbo spin-echo (TSE) pulse sequence with a repetition time (TR) of 4050 msec, echo time (TE) of 99 msec, flip angle of 170°, 210 mm field of view (FOV), and 512 × 336 matrix. Forty axial slices were acquired with a thickness of 4 mm (no gap) and an in-plane resolution of 0.47 × 0.47 mm. These structural images were obtained to register and align the functional images with an anatomical reference. Functional T2*-weighted images reporting blood oxygenation level-dependent (BOLD) signals were acquired at the same 40 slice locations, using gradient-echo echo-planar images with a TR of 2500 msec, TE of 27 msec, flip angle of 82°, FOV of 240 mm, and an acquisition matrix of 64 × 64. Each functional image comprised a brain volume of 40 axial slices with 3 mm thickness (1-mm gap) and an in-plane resolution of 3.75 × 3.75 mm. All images were acquired with slices positioned parallel to the anterior commissure–posterior commissure line. All participants completed four runs of 380 sec each, yielding 152 time points per run.

### Statistical analysis

#### Behavioral analyses

The primary measures of performance on the behavioral task were RT and accuracy of responses over the four conditions: (i) congruent flanker following non-reward cue; (ii) congruent flanker following reward cue; (iii) incongruent flanker following non-reward cue; and (iv) incongruent flanker following reward cue. A two-way repeated measures analysis of variance (ANOVA) with cue (reward vs. non-reward) and flanker (congruent vs. incongruent) as within-subjects factors was used to test the interaction of reward with RT and accuracy. We also conducted post hoc analyses of RT in relation to the preceding reward outcome by creating three additional variables: RT1 for trials that followed expected reward outcomes, RT2 for trials that followed surprising non-reward outcomes, and RT3 for rewards that followed punishment outcomes. These variables were analyzed using a one-way ANOVA. The alpha level for these analyses was set at *P* < 0.05.

#### fMRI analyses

Image processing was conducted using statistical parametric mapping (SPM5; Wellcome Department of Imaging Neuroscience, London, U.K.). Standard SPM preprocessing of the functional time series was performed individually for each subject. The functional scans were slice time-corrected, realigned to the first volume to correct for interscan motion, coregistered to the T2 image, normalized to a standard template (Montreal Neurological Institute), and spatially smoothed with an 8 × 8 × 8 mm^3^ full-width at half-maximum (FWHM) Gaussian kernel.

First-level analyses were conducted individually for each participant with a general linear model (GLM) to quantify the relationship between event-related BOLD signals and regressors encoding neural responses to trial factors. In other words, each trial (with cue and outcome components) was modeled as a single (compound) event and response components were modeled in terms of putative processing components elicited by the task design. Specifically, regressors were created by convolving a train of delta functions that represented the individual trial types with the canonical hemodynamic response function, composed of two gamma functions (Friston et al. 1998). The six-movement estimates from the realignment procedure were entered as covariates of no interest (Johnstone et al. 2006). The design matrix comprises nine regressors of interest: six for cue (reward vs. non-reward) and flanker-type (congruent or incongruent) effects and three for outcome-related effects. The six-cue regressors consisted of two regressors modeling the main effect of reward versus non-reward cue over all trials (i.e., anticipation), and an additional four regressors to model the effects of reward cue and target congruence (and their interaction) for correct (and nonpunishment) trials. The three outcome-related effects were reward following reward cue, non-reward following reward cue, and non-reward following non-reward cue. Due to high accuracy of performance and few punishment outcomes (i.e., not enough events were present to generate a composite image), we did not introduce a punishment regressor. This event-related analytic approach is optimal for this particular task design because the presentation of cues and flankers are orthogonal.

The main effect of reward anticipation was tested with appropriate linear contrasts of the parameter estimates for the reward cue minus non-reward cue. The neural substrate of cognitive conflict was tested by contrasting incongruent versus congruent flankers (i.e., the main effect of congruency in correct trials). In addition, the interaction between reward anticipation and conflict resolution in correct trials was tested by contrasting incongruent targets minus congruent targets preceded by reward cues versus non-reward cues. The reward outcome effects were tested with two contrasts: the effect of reward per se was summarized by subtracting the expected non-reward from the expected reward. The effect of surprising non-reward was assessed by subtracting the expected non-reward outcome from the surprising non-reward outcome. Note that the term “reward outcome” is used to refer to the particular outcome for each individual trial – not to the reward outcome of the preceding trial. Also we did not analyze penalty or punishment effects because of the small number of incorrect (or slow) responses (see Table 2). In this sense, the incentive effects are driven largely by the (fictive) reward outcomes – noting that the actually monetary recompense for participating in the study was established in advance and was the same for all subjects.

**Table 2:** Behavior results

	Trial type			
	
	Congruent target		Incongruent target	
		
Variables	Reward cue	Non-reward cue	Reward cue	Non-reward cue
Reaction time (mean, SD)	518.4 (±83.9)	523.4 (±96.7)	567.1 (±95.2)	584.8 (±103)
Accuracy (%)	98.4	98.8	96.6	97.5

The two-way ANOVA revealed: RTs were significantly different for reward versus non-reward cues (*F*_1,15_ = 5.900, *P* = 0.028). RTs were significantly longer for incongruent relative to congruent flankers (*F*_1,15_ = 92.032, *P* < 0.001). Accuracy was not significantly different among different trial types.

The ensuring contrast images for each participant were entered into second-level random-effects group analyses, using one sample *t*-tests to produce statistical parametric *t*-maps (SPMs) testing for regionally specific effects. The fMRI results are reported at a corrected significance level of *P* < 0.05 using a Monte Carlo correction with cluster size threshold of 85 (2 mm^3^).

Group-level interaction effects between anticipation (reward vs. non-reward) and conflict (congruent vs. incongruent) were determined by a 2 × 2 repeated measures ANOVA. We illustrated the significant interaction effects plotting the magnitudes of the effects in each region obtained with an 8-mm radius sphere centered on the peak voxel of target-related activity in each region. Interaction effects were tested within volumes defined by the (orthogonal) main effects of anticipation. The use of orthogonal localizing contrasts protects against biased sampling (Friston et al. 2006).

## Results

### Behavioral results

**Figure 2:**
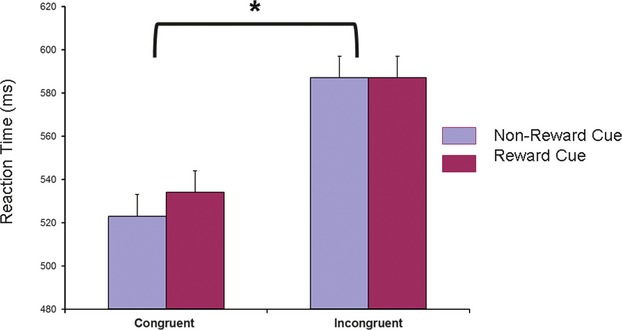
Behavior results.

There was a significant main effect of conflict on RT, with RTs significantly longer for incongruent than congruent flankers (Table 2, *F*_1,15_ = 92.258, *P* < 0.001). Similarly, there was a significant main effect of anticipation (*F*_1,15_ = 5.900, *P* < 0.028). However, there was no interaction between anticipation and conflict (*F*_1,15_ = 3.226, *P* = 0.93) (Table 2, Fig. 2). Although response accuracy was higher for congruent (98.6%) versus incongruent flankers (96.9%), these differences were not significant. Post hoc analyses showed that RT2 (mean = 544.30 msec, SD = 92.58 msec) was significantly shorter than RT1 (mean = 556.34 msec, SD = 107.32 msec, *P* = 0.038), and that RT3 was the longest (mean = 622.97 msec, SD = 215.40 msec).

### Neuroimaging results

#### Reward anticipation

**Figure 3:**
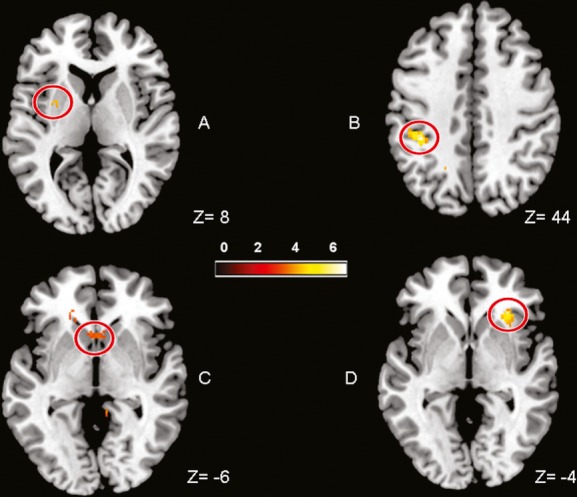
Activation during reward components of the ACR task. Statistical parametric maps in axial views showing significant blood oxygenation level-dependent (BOLD) signal changes. (A) BOLD signal increase in the left putamen generated by the reward–non-reward cue contrast. (B) BOLD signal increase and the left parietal cortex generated by the reward–non-reward cue contrast. (C) BOLD signal decreases in the left ventral striatum generated by surprising non-reward–expected non-reward outcome contrasts. (D) BOLD signal increase in the right insula generated by the surprising non-reward–expected non-reward outcome contrast. The figures were thresholded at *P* < 0.05 (corrected); the color bar indicates color-coded significance of the *t*-test values.

Contrasts for reward minus non-reward cues showed significant activation in components of the attentional network, including the right superior parietal cortex, the inferior occipital cortexes bilaterally, the left lingual gyrus, the left thalamus, and the left putamen (Table 3, Fig. 3).

**Table 3:** Regions showing activation during the Reward Anticipation (reward minus non-reward cue) component of the ACR task

Region	Side	MNI coordinates			*Z*	Voxels
Thalamus	Left	−18	−10	8	3.70	273
Lingual cortex	Left	−12	−78	−10	3.66	193
Inferior occipital cortex	Left	−42	−74	−4	2.90	317
Inferior occipital cortex	Right	46	−74	−2	4.30	669
Superior parietal cortex	Left	−38	−38	44	3.66	625
Middle frontal gyrus	Right	42	52	14	3.50	273
Putamen	Left	−30	2	8	3.50	273

#### Cognitive conflict

**Figure 4:**
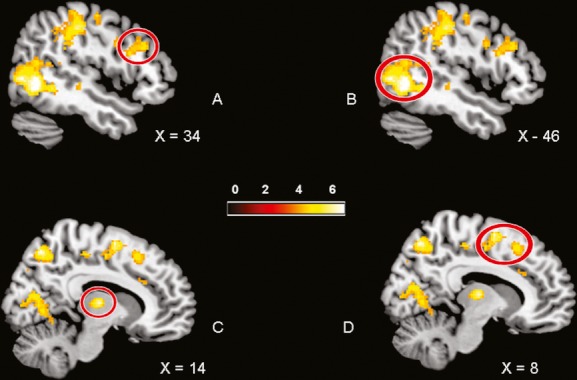
Activation during cognitive conflict component of the ACR task. (A) BOLD signal increase in the right inferior frontal gyrus generated by incongruent–congruent flanker contrasts. (B) BOLD signal increase and the right middle temporal cortex generated by incongruent–congruent flanker contrasts. (C) BOLD signal increase in the left thalamus generated by incongruent–congruent flanker contrasts. (D) BOLD signal increase in the left supplemental motor area generated by incongruent–congruent flanker contrasts. The figures were thresholded at *P* < 0.05 (corrected); the color bar indicates color-coded significance of the *t*-test values.

The incongruent minus congruent flanker contrast showed robust activation in a distributed corticothalamic network including the right ACC, right primary motor cortex, the supplemental motor and somatosensory association cortices bilaterally, as well as the right middle frontal gyrus (MFG) and right thalamus (Table 4, Fig. 4).

**Table 4:** Regions showing activation during the Cognitive Conflict component of the ACR task

Region	Side	MNI coordinates			*Z*	Voxels
Supplemental motor cortex	Right	8	20	48	3.70	99
Fusiform gyrus	Left	−42	−46	−20	2.60	1500
Inferior frontal cortex	Right	34	26	−6	3.40	1500
Somatosensory association cortex	Right	10	−66	54	3.90	3218
Somatosensory association cortex	Left	−24	−68	52	3.60	357
Middle frontal gyrus	Right	36	22	26	4.44	268
Thalamus	Right	14	−14	10	4.35	227
Superior parietal cortex	Right	34	−38	46	4.50	3218
Middle temporal cortex	Right	48	−68	0	4.30	1500

#### Expected reward

**Figure 5:**
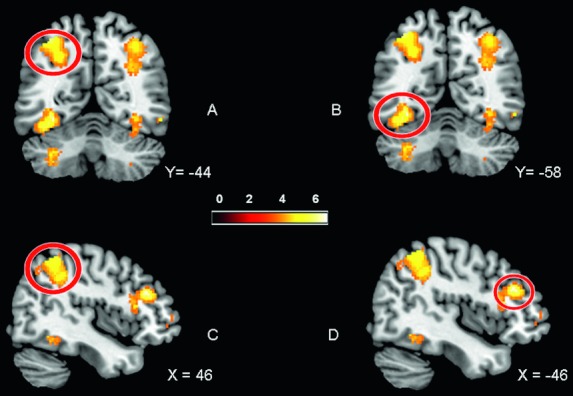
Activation during expected reward component of the ACR task. (A) BOLD signal increase in the left parietal cortex generated by reward–expected non-reward outcome contrasts. (B) BOLD signal increase and the left lingual cortex generated by reward–expected non-reward outcome contrasts. (C) BOLD signal increase in the right parietal cortex generated by reward–expected non-reward outcome contrasts. (D) BOLD signal increase in the right inferior frontal gyrus generated by reward–expected non-reward outcome contrasts. The figures were thresholded at *P* < 0.05 (corrected); the color bar indicates color-coded significance of the *t*-test values.

The expected reward (i.e., reward outcome that followed a reward cue and correct target response) minus expected non-reward (i.e., neutral outcome that followed a non-reward cue and correct target response) contrast was associated with activation in the inferior parietal, fusiform, and occipital cortices bilaterally, and the right inferior temporal cortex (Table 5, Fig. 5).

**Table 5:** Regions showing activation during the Expected Reward component of the ACR task

Region	Side	MNI Coordinates			*Z*	Voxels
Inferior parietal cortex	Right	46	−44	54	4.31	1002
Inferior parietal cortex	Left	−36	−54	48	3.85	643
Superior parietal cortex	Left	−28	−64	54	4.21	643
Superior occipital cortex	Right	28	−64	−24	3.85	1002
Middle occipital cortex	Left	−26	−70	26	3.85	643
Middle frontal gyrus	Left	−46	28	32	3.60	2429
Fusiform gyrus	Right	34	−58	−8	3.90	1089
Fusiform gyrus	Left	−40	−58	−16	3.90	1714

#### Surprising non-reward

The contrast of surprising non-reward (i.e., non-reward outcome following a reward cue and a correct target response) minus expected non-reward elicited activation in the insula bilaterally and deactivation bilaterally in the ventral striatum (Table 6, Fig. 3).

**Table 6:** Regions showing activation during Surprising Non-Reward component of the ACR task

Region	Side	MNI coordinates			*Z*	Voxels
Insular cortex	Right	33	26	−4	4.80	329
Insular cortex	Left	−42	12	8	3.60	182
Thalamus	Right	3	−10	2	3.55	106
*Deactivation*						
Ventral striatum	Left	−4	18	−6	3.50	97
Ventral striatum	Right	8	18	−4	3.50	97

#### Reward anticipation by cognitive conflict interaction

**Figure 6:**
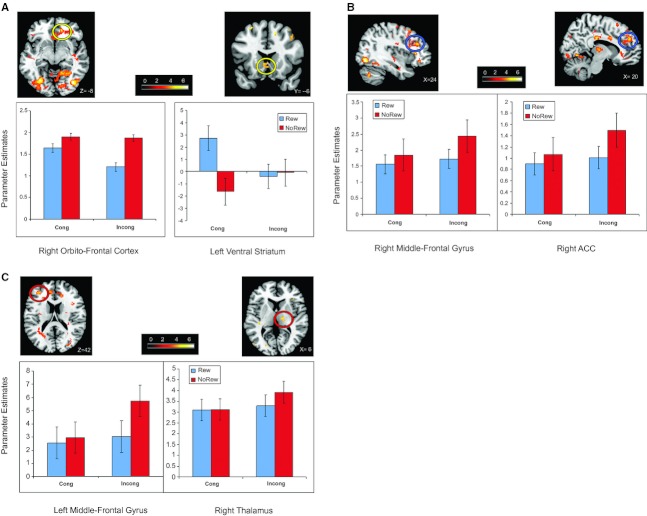
Anticipation × Cognitive Conflict interactions estimated percent change in the BOLD signal during congruent and incongruent flankers of the ACR task in relation to the preceding cue (i.e., reward vs. non-reward) in (A) right orbitofrontal gyrus and left ventral striatum, and (B) right middle frontal gyrus and right anterior cingulate cortex, and (C) left middle frontal gyrus and the right thalamus. The SPMs were thresholded at *P* < 0.01; the color bar indicates color-coded significance of the *t*-test values.

Regions that exhibited significant interactions between anticipation (reward vs. non-reward cue) and conflict (congruent vs. incongruent targets) are presented in Table 3. Parameter estimates in these regions showed two distinct patterns of signal change that were linked to the purported functions of the regions (i.e., ventral striatum and OFC – consistent with their functions as parts of the reward system; and thalamus, ACC, and middle frontal gyri – consistent with their functions within the attentional system). Activation during targets that followed reward cues was higher for congruent than incongruent targets in the ventral striatum and the OFC, but there was no difference in activation between the two types of targets in the thalamus, ACC, and MFG bilaterally. Activation during targets that followed non-reward cues were higher for incongruent than congruent targets in the thalamus, ACC, MFG bilaterally, and ventral striatum, but not different in the OFC (Table 7). Thus, cognitive conflict elicited greater activations, but only in the absence of reward anticipation. In the presence of reward anticipation, congruence or conflict-dependent differences were diminished in the thalamus, ACC, and MFG bilaterally and reversed in the OFC and the ventral striatum (Fig. 6).

**Table 7:** Regions showing activation during Anticipation × Cognitive Conflict interactions

Region	Side	MNI coordinates			*Z*	Voxels
Caudate/ventral striatum	Left	−2	18	−6	2.60	208
Orbitofrontal gyrus	Right	14	42	−8	3.20	346
Anterior cingulate gyrus	Right	14	32	20	2.80	345
Thalamus	Right	12	−16	6	3.50	633
Middle frontal gyrus	Right	36	34	24	3.35	604
Middle frontal gyrus	Left	−46	12	42	3.25	98

## Discussion

### General discussion

Our results demonstrate that the reward-related components of the ACR activated brain regions in both the reward and attentional networks; however, there was a dissociation between the effects of reward and non-reward cues. Specifically, reward cues and obtaining expected rewards activated the superior and inferior parietal and the inferior occipital cortices bilaterally and the right inferior temporal cortex, all regions within the attentional network. In contrast, surprising non-reward (i.e., when non-reward was given for correct responses following reward cues) affected regions of the reward system – as evidenced by increased activation in the bilateral insula and deactivation in the ventral striatum. As hypothesized, cognitive conflict – produced by incongruent targets – activated the ACC and the primary and supplementary motor cortices. Interaction effects were seen in components of the reward and attentional systems to congruent versus incongruent targets, in relation to anticipation (reward vs. non-reward cues). Activations were greater for incongruent (conflict) relative to congruent (no conflict) trials during targets that followed non-reward cues, suggesting that in the absence of reward incentives, the differential activation in attentional networks can be explained by the congruency effect and associated cognitive demand. However, incongruent (conflict) targets that followed reward cues were associated with less activation in the ventral striatum and OFC suggesting that reward cues diminished the conflict-dependent activation in the reward system.

In order to understand the patterns of activation elicited by the different conditions in the ACR task, it is important to examine the relationships among the components of the task, and to understand the possible psychological processes associated with these relationships. First, a key difference between the ACR task and other reward paradigms (Knutson et al. 2000; Bjork and Hommer 2007) is that the ACR task presents a fixed amount of reward (e.g., $1) and two levels of reward incentive – reward (e.g., $1) and non-reward ($0). In addition, the ACR task is a performance-dependent task with several dimensions of demand: (i) demand for fast responses and (ii) demand for accurate responses with both congruent versus incongruent (i.e., easy vs. difficult) flanker trials. In this respect, the ACR task furnishes a high probability for negative (over positive) outcomes. For example, only 50% of all the reward cue trials (which in turn represent 50% of all trials) are rewarded if the subject performs with 100% accuracy. Therefore, perfect performance will entail fixed rewards of $1 in 25% of all trials and produce violation of reward expectation (e.g., surprising non-reward) in another 25% of trials. Therefore, the ACR task may be experienced as a task with high attentional demand associated with limited opportunities for rewards. As such, the ACR task seems well suited for assessing psychological reactions related to both reward processing in the context of high cognitive demand, as well as violation of reward expectations. In this study, we deliberately suppressed contextual effects of accumulated outcomes by telling all the subjects in advance that only 50% of reward trials would be rewarded. Furthermore, the high level of accuracy (about 97%) precluded any contextual effects of punishment on subject performance. In addition, the results from the post hoc behavioral analyses showing that the participants responded fastest following surprising non-reward trials suggest that they remained motivated to obtain rewards through the duration of the task and that the overall context of the task as a task with limited opportunities for rewards did not have demoralizing effect on the performance. As we did not analyze penalty or punishment effects due to small number of incorrect (or slow) responses (see Table 2), the incentive effects are driven largely by the (fictive) reward cues – noting that the actual monetary recompense for participating in the study was established in advance and was the same for all subjects.

### Effects of cognitive demands on reward processing

The cue by target interactions found in the left ventral striatum indicates that participants activated this region more during targets with the highest probability of furnishing reward (i.e., congruent or “easy” flankers that followed reward cues, Fig. 6A), suggesting that participants may have experienced these trials as the most rewarding. This finding is in line with reports from others (Botvinick et al. 2009) demonstrating that the activation in the ventral striatum may be inversely influenced by the degree of mental effort required to obtain individual rewards. Similarly, congruent flankers that followed reward cues produced higher activation in the right OFC, a region that provides reward-related feedback. It is possible that deactivation in components of the reward network during incongruent flankers (i.e., “difficult” trials) was attributable to offering the same amount of reward (e.g., $l) for all reward cues, even when the need for attentional effort remained high. This provides a rationale for why rewards that demanded less attentional effort may have been experienced as the most rewarding, consistent with the observed elevated striatal and OFC activation during congruent (easy) flankers that followed reward cues (Fig. 6A). Similarly, it is plausible that trials requiring high cognitive demands (i.e., having to sort through incongruent stimuli) may have been experienced as too difficult in relation to the expected reward. This last suggestion is in line with findings showing that money incentives may hamper performance on cognitive tasks (Padmala and Pessoa 2010). However, the proposition that reward incentives may not have the purported uniform effect of increasing motivation (and, by extension, cognitive effort) but rather may reduce cognitive effort during specific (i.e., more difficult) components of a cognitive tasks needs to be explored further.

### Effects of motivation on cognitive control

The primary effects of reward cue were registered in components of the attentional network. In addition, we registered activation in the left putamen (i.e., motor area), possibly associated with preparation for action and indicating that the reward cue was motivating subjects to respond to the task. These results suggest that the reward cues in this study were experienced both as a signal to pay attention and to motivate one's actions to obtain reward. Considering the high demand for correct responses during the ACR task, it is plausible that participants may not have been motivated by the monetary value of the cues (e.g., one “virtual” dollar) but by the desire to respond correctly. The positive effect of reward incentives on the preparatory stage of task performance has been described in other paradigms (e.g., task-switching [Savine and Braver 2010]). Given the high probability for negative outcomes in the reward condition of the ACR, these cues may have elevated the level of attention preceding the target in order to optimize the positive outcomes (as money wins were possible only after reward cues).

The interaction analyses showed that the participants generate higher activation during targets with non-reward potential and higher probability for punishment (i.e., incongruent “difficult” flankers following non-reward cues, Fig. 4). Therefore, the effect of the reward cues on the activation of ACC, thalamus, and MFG was to reduce the activation during the more difficult incongruent flanker. These findings are in line with a recent report that reward incentives may diminish conflict-associated activation in attentional networks (Padmala and Pessoa 2011). In this study, reward incentives appeared to enhance the activity of the attentional system when preparing to initiate a response, and to diminish activation in components of the attentional system in response to the “easy” congruent stimuli, all of which could result in performance optimization.

Alternatively, the experience of lack of reward as a potential “motivator” in the non-reward cue trials may have been more salient for subjects than the anticipation of reward incentive. In other words, the motivation to avoid a $1 penalty may have be more salient than the anticipation of winning $1 in reward trials (which occurs only in 50% of the trials), and could have been driving the greater activations in attentional regions in the non-reward cued trials versus reward cued trials.

### Effects of surprising non-reward

The effects of surprising non-reward on the ACR may be of particular interest, as this topic has received limited attention in the literature. The observed robust activation of the bilateral frontal insula during the surprising non-reward outcomes of the ACR task may reflect emotional experiences and/or negative arousal associated with the higher uncertainty about winning a reward during this particular task condition. This thesis is supported by reports that activation of the frontal insula may be linked to choosing “safe” strategies following punishment and the mental representation of affective reaction to reward outcomes (Paulus et al. 2003), as well as to emotional experiences of uncertainty about possible reward outcomes (Linke et al. 2010). The observed bilateral deactivation of the ventral striatum in association with unexpected non-reward (e.g., violation of reward anticipation) is in line with results from several other studies that have documented deactivation of the ventral striatum associated with negative outcomes (Knutson et al. 2004; Botvinick et al. 2009). It is hypothesized that this type of deactivation may be linked to monitoring of reward outcomes (gains vs. losses). If true, then it stands to reason that during a task that places high demands for correct performance and offers limited opportunities to obtain rewards, negative outcome trials will robustly engage the ventral striatum as it purportedly tracks the performance and related outcomes.

In summary, this study demonstrates a dissociation of the effects of motivation (reward cues) and outcomes in the context of a paradigm with high demand for attention and cognitive control. The experience of loss seems to have been more salient for the subjects than the experience of reward incentives, as the violation of reward expectations consistently engaged the insula and the ventral striatum, whereas the reward cues activated robustly the components of the attentional network and did not elicit strong activation in brain regions associated with motivation.

### Limitations

Two aspects of the ACR task potentially limit the interpretation of our results with regard to reward mechanisms. Although the ACR is a performance-dependent task, the attainment of reward during the task was not linked to variation in the monetary compensation given to subjects, which may explain the less robust activation of the reward system during task performance. Furthermore, we did not observe any significant behavioral effects of reward on RT or accuracy, which further suggests the absence of graded reward incentives. It remains to be determined if our findings would be the same if we tied subject compensation to money earned or lost during the task.

## Conclusions

The results of this study indicate that reward incentives and outcomes are associated with activation of brain regions that are often considered to mediate to attentional and reward functions. More importantly, a subset of these regions, including the right medial frontal gyrus, thalamus, ACC, left ventral striatum, and OFC, exhibited significant reward cue by target interactions. These results suggest that potentially rewarding trials (e.g., reward cues) are associated with lower activation in attentional networks during following targets, possibly due to increased efficiency. In contrast, non-reward trials with high probability for money loss (e.g., non-reward cue followed by incongruent/most difficult targets) appear associated with higher activity in attentional networks indicating possible compensatory efforts to avoid punishment. In addition, these trials were also associated with lower activity in regions of the motivational system, suggesting that they may be experienced as less rewarding.

## Supporting Information

**Table S1.** Instruction for subjects.
