# Supplementary material for: Effects of motivation on reward and attentional networks: an fMRI study
Source: Brain Behav. 2012 Sep 23;2(6):741–53. doi: 10.1002/brb3.80 (PMC3500461; doi:10.1002/brb3.80)
Supplement: Supplementary file 1 [file brb30002-0741-SD1.doc]

**Supplemental Materials**

**Table S1.** Instruction for subjects

**Page 1**

Welcome to the reward experiment!

You will see cues followed by targets, and then by rewards in this study. If the cue is in blue color, you have a 50% chance to get $1 if you make a fast and correct response to the target. If you see a cue in yellow color, there will be no reward.

The target is a central arrowhead pointing to the left or the right. The central arrowhead is surrounded by two other arrowheads on both the left and right sides, either pointing in the same direction or in the opposite direction as the central arrowhead. There are four target types:

left right left right

Only the central arrowhead is the target. Press your left index finger when the central arrowhead points to the left and press your right index finger when the central arrowhead points right. Please respond to the target everytime you see one. You need to make your response as quickly and accurate as possible.

The reward will be: in green color, and no reward will be in light blue color.

**Page 2**

The chance of getting a reward is 50% for the reward trials if you make a correct and fast response to the target. For the non-reward trials, you also need to make your response as quickly and accurate as possible. For both reward and non-reward trials, if your response is incorrect or slow, you will be punished and will see in red color and you will lose $1.

Please do not move your head cross the whole experiment.

Reminder: left index finger for a target pointing to left and right index finger for a target pointing right.

There will be four blocks for the task. Each black takes about 6 minutes.

Your total reward will be displayed at the end of each block.

Please keep your fixation on the crosshairs and make sure you respond as quickly and accurately as possible.
